# Prolonged Transient Global Amnesia: Part of the Clinical Spectrum or a Separate Disease Entity?

**DOI:** 10.1177/19418744231184120

**Published:** 2023-06-20

**Authors:** Heather Y.F. Yong, Carlos R. Camara-Lemarroy

**Affiliations:** 1 Department of Clinical Neurosciences, 70401 University of Calgary, Alberta, Canada; 2 70401 Cumming School of Medicine, University of Calgary, Calgary, Alberta, Canada

**Keywords:** Transient global amnesia, TGA, imaging, prolonged amnesia

## Abstract

**Background:**

Transient global amnesia (TGA) is the prototypical neurologic disease for acute-onset reversible amnesia. It is currently defined by resolution of symptoms within 24-hours. In this case report we describe an atypical case of prolonged TGA, emphasizing our current lack of knowledge surrounding this disease entity and its pathophysiology.

**Results:**

A 66-year old female presented acutely with profound anterograde amnesia and variable retrograde amnesia with no inciting event. A thorough workup to exclude alternative causes of amnesia (including computed tomography angiogram and electroencephalogram) was normal. Her magnetic resonance imaging was consistent with TGA, with punctate diffusion restriction changes bilaterally in the hippocampi. She was also mildly hypoxemic with no discernible cause. She was ultimately diagnosed with TGA although her diagnosis remains controversial as her symptoms persisted for 72-hours.

**Conclusion:**

Our patients clinical and imaging features (apart from her protracted time-course and hypoxemia) were in keeping with a diagnosis of TGA. The association of hypoxemia, COVID-19, obstructive sleep apnea, and the development of TGA remains to be elucidated. Although the underlying pathophysiology for TGA is unknown several mechanisms have been postulated including cortical spreading depression and reversible hypoxic-ischemic injury. The time course for symptom resolution, could be an important clue in discerning the pathophysiology of TGA on an individual basis. Importantly, a clinician should not be deterred by amnestic symptoms lasting >24-hours, if the patients clinical/radiologic presentation is consistent with TGA.

## Introduction

Acute-onset amnesia is a dramatic neurologic presentation with a broad differential including cerebrovascular insult, transient epileptic amnesia, psychogenic/post-traumatic disorder, encephalopathy, and toxin/drug ingestion^1^. The prototypical disease with this distressing (yet short-lived) presentation is transient global amnesia (TGA). TGA is defined by profound anterograde amnesia with variable retrograde amnesia lasting up to 24-hours (Table 1).^1,2^ Herein we describe an atypical TGA case prompting questions about our current diagnostic criteria and the underlying pathophysiological mechanisms that contribute to TGA.

**Table 1.** Diagnostic criteria for transient global amnesia compared to our patients presentation. Known diagnostic criteria by Hodges and Warlow (1990)^2^ and adapted by Miller and Butler (2022)^1^ is in the left column, while our patients’ clinical characteristics are in the right column. EEG = electroencephalogram; h = hour.

Transient global amnesia criteria^1,2^	Index case
1. Witnessed attack	✓ Witnessed by husband
2. Anterograde amnesia (evidenced with repetitive questioning)	✓ Profound anterograde amnesia, mild retrograde amnesia
3. Cognitive impairment limited to amnesia	✓ No deficits in other cognitive domains
4. No clouding of consciousness or loss of personal identity	✓ No delirium or psychiatric history
5. No focal neurologic signs or symptoms	✓ Normal neurologic exam, no other deficits on neuroimaging
6. No epileptic features	✓ No history of epilepsy, normal EEG
7. No recent head injury or active epilepsy	✓ No history of epilepsy, normal EEG, normal neuroimaging
8. Resolution of symptoms within 24h	✗ **Symptoms lasting >72h**

## Case Description

A 66-year old female with a history of hypertension, dyslipidemia, and hypothyroidism presented to the emergency department with acute-onset amnesia without a clear emotional or psychological stressor. The patient was not formally cognitively assessed but on bedside neurologic examination there were no focal deficits other than profound anterograde amnesia (0/3 with delayed recall, primarily episodic memory with intact semantic memory), mild retrograde amnesia, and perseveration with repetitive questioning. The patient had no broader cognitive deficits in the domains of language (normal fluency, ability to follow 1-3 step commands, and name low/high frequency objects), apraxia, and executive functioning (tested with Luria hand sequencing). There were no signs of delirium or difficulties with complex attention when doing serial 7’s and WORLD bedside testing.

She had no history of psychiatric disease and there was no evidence of acute drug intoxication (including a normal urine toxicology screen). Her biochemical profile was normal (including B12 levels, and liver/kidney/thyroid function). Brain and neck computed tomography (CT)-angiogram did not reveal cerebrovascular insult or a structural lesion. Her electroencephalogram was normal with no focal slowing, epileptiform discharges, or seizures.

**Figure 1.**
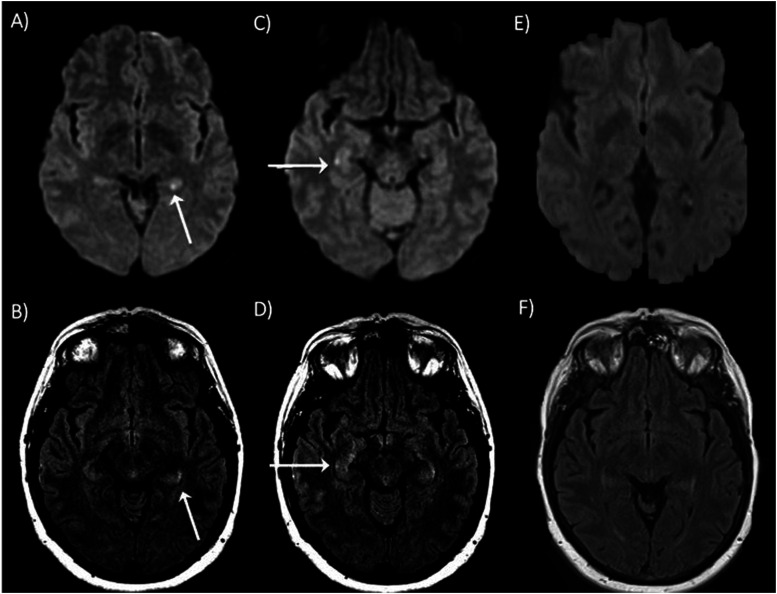
Diffusion weighted imaging (DWI) changes in a patient with suspected transient global amnesia. On initial brain magnetic resonance imaging (MRI) 2 punctate foci of restricted diffusion were seen in the hippocampi bilaterally. The left-sided DWI lesion was in the lateral aspect of the hippocampal head (A), with corresponding hypointensity on apparent diffusion coefficient (ADC, not shown in this figure), and hyperintensity on fluid attenuated inversion recovery (FLAIR, B). A right-sided DWI lesion was positioned more posteriorly towards the hippocampal body (C), with corresponding hypointensity on ADC, and hyperintensity on FLAIR (D). A repeat MRI 6-months later showed complete resolution of the punctate foci of diffusion restriction (E, F).

Brain magnetic resonance imaging (MRI) 2-days from symptom onset revealed punctiform diffusion weighted imaging (DWI) restriction changes bilaterally in the medial temporal lobes (Figure 1A-D) with corresponding hypointensity on apparent diffusion coefficient (not shown). DWI hyperintensity was noted in the left posterior hippocampal body (Figure 1A), with corresponding fluid attenuated inversion recovery imaging (FLAIR) hyperintensity (Figure 1B). Diffusion restriction and FLAIR hyperintensity was also noted in the right lateral aspect of the hippocampal head (Figure 1C, D). Her history, lack of other focal neurologic deficits, normal extended amnestic investigations, and classic imaging findings^1,3,4^ were felt to be consistent with a diagnosis of TGA.

In addition to her acute amnesia she was found to be mildly hypoxemic for an unknown duration. She tested negative for COVID-19 and respiratory syncytial viruses on serial testing, with a normal white blood cell count, D-dimer, troponin, and arterial blood gas. Serial chest X-Rays revealed slightly prominent lung markings with no evidence of collapse or consolidation. A CT-chest did not reveal an underlying pathology for her hypoxemia/evidence of thrombus, and pulmonary function tests were within normal limits. She was weaned off oxygen prior to discharge.

Her amnesia rapidly improved without treatment; however, she remained intermittently amnestic for 72-hours post symptom onset which is not in keeping with a diagnosis of TGA (Table 1).^1,2^ At follow-up she had returned to her cognitive baseline, with complete resolution of her clinical symptoms and MRI findings (4-months from symptom onset; Figure 1E and F).

## Discussion

Our patients clinical and imaging features were consistent with current criterion for TGA (Table 1)^2^ with dense anterograde amnesia (and variable retrograde amnesia), bilateral punctate hippocampal DWI lesions, and lack of alternative causes of acute amnesia. Brain MRI in TGA can often be normal, however bilateral/unilateral punctate DWI lesions (with predilection for the CA1 hippocampal region) are noted in up to 84% of patients.^1,3,4^ Typically these changes appear *after* the amnestic period has resolved, with the highest detection rate 48-hours after symptom onset.^1,3^ This differs from our patient, whose lesions were noted in the acute symptomatic phase. DWI findings typically resolve within 6-12 months,^5^ similarly to our patient.

Controversially, the protracted timeframe of her symptom resolution was not in keeping with a TGA diagnosis (72-hours instead of 24-hours;^2^ Table 1). Dramatic amnesia in TGA typically resolves within a few hours, although a handful of case reports/series have noted prolonged cognitive deficits.^6-12^ In one case-report profound amnesia lasted up to 8-days,^8^ and in a retrospective observational study of 185 TGA patients subtle reductions in cognition lasting longer than 24-hours were noted (as measured with the mini-mental status examination, verbal long-term memory, and executive functioning).^9^

Although the pathophysiology for TGA is unknown, the symptomatic timeframe may implicate multiple underlying processes. Several pathophysiological mechanisms have been postulated in TGA including cerebrovascular insult with small vessel abnormalities, hypoxic-ischemic changes with venous congestion in memory relevant structures, or cortical spreading depression in association with migraines/epilepsy/enhanced physiological or emotional stress.^1,3^ A shorter TGA duration (<1-hour) could favor cortical spreading depression, while a longer TGA duration could be more in keeping with transient hypoxic-ischemic injury or even cerebral ischemia. Regardless of the underlying mechanism it is important to not strictly rely on the 24-hour criterion when the clinical/radiologic presentation is consistent with TGA.

Further adding to the complexity of our patients’ case was her hypoxemia. This is not a known complication of TGA although a recent case report highlighted TGA as a possible first manifestation of COVID-19 and hypoxemic respiratory failure.^13^ Further, a study by Buratti et al (2017) highlighted a higher prevalence of obstructive sleep apnea (OSA) in TGA patients.^14^ In this study the prevalence of OSA in TGA patients was 44.8% compared to 13.8% in the general population (P = .02) for an odds ratio of 5.078 (95% confidence interval 1.405-18.345; P < .05).^14^ Our patient did not have known OSA, however it is tempting to postulate an association between OSA-induced hypoxic-ischemic injury and TGA. Certainly sleep and memory are closely related, and subtle cortical damage in areas of memory processing have been noted in OSA patients.^14-16^ Further studies on the association between systemic hypoxia/COVID-19/OSA/TGA are worth considering.

## Conclusion

In summary, this case highlights a patient with typical clinical/imaging findings of TGA although her protracted symptomatology and hypoxemia made the diagnosis controversial. It is difficult to discern whether a protracted course of amnesia points towards a different pathophysiological mechanism for the development of TGA. Regardless, it is important for the clinician to not strictly rely on the 24-hour criterion if a patients presentation is in keeping with TGA as a timely diagnosis can help avoid extensive investigations, misdiagnosis of ischemia or irreversible pathology, and reassure patients and their relatives of the benign character of this dramatic disorder.
